# From Evidence to Strategic Insight: Preliminary Model Development and Validation for Exploring Potential Changes and Enhancing Nursing Work Performance

**DOI:** 10.1155/jonm/6911579

**Published:** 2026-10-17

**Authors:** Haizhe Jin, Junnan Zhou, Kaifang Ren, Jianjun Cheng, Zhibin Xiao, Jiahao Zhang, Zibo Gong

**Affiliations:** ^1^ Department of Industrial Engineering School of Business Administration Northeastern University #195, Chuangxin Road, Hunnan-Qu Shenyang 110167 China neu.edu.cn; ^2^ Dermatology Department Dongguan Hospital of Traditional Chinese Medicine #3, Songshanhu Road Dongguan 523000 China dg-tcm.com; ^3^ Emergency Medicine Department Zhongxin Huizhou Hospital #346, Zhongkai Road Huizhou 516000 China; ^4^ School of Business Administration Northeastern University No. 195, Chuangxin Road, Hunnan District Shenyang 110167 China neu.edu.cn; ^5^ Department of Radiology Shengjing Hospital of China Medical University No. 36, Sanhao Street Shenyang Liaoning 110004 China cmu.edu.cn

**Keywords:** causal relationship, health care, model simulation, performance improvement, system dynamics

## Abstract

**Aim (s):**

To develop a dynamic analytical framework integrating nursing work performance types and their influencing factors and explore potential changes under different management strategies.

**Background:**

Nursing work performance directly influences healthcare quality and patient safety. However, existing research has not yet established an analytical framework that systematically integrates nursing work performance types, their influencing factors, and the dynamic mechanisms.

**Method (s):**

A systematic literature review and system dynamics (SD) approach were applied. Expert evaluation and parameter perturbations were used to assess the structural rationality and stability of the model.

**Results:**

Of 2289 records identified, 1619 unique records were screened, and 47 studies were included. Five types of nursing work performance were extracted and synthesized: Quality Safety, Rescue Efficiency, Patient Satisfaction, Job Satisfaction, and Process Optimization. Seven organizational factors were identified: Cultural Climate, Leader Attitude, Supervisory Governance, Incentives and Support, Resource Allocation, Task Allocation, and Education and Training. Three individual factors were identified: Physiological State, Professional Competence, and Psychological State. Based on these findings, an SD model was constructed using causal loop and stock‐and‐flow diagrams to represent the relationships among these components. Expert evaluation indicated that the model structure was generally reasonable and that the model outputs maintained broadly consistent directional trends under parameter perturbations and simulation scenarios. Finally, scenario simulations were conducted to analyze potential changes in nursing work performance under different management strategies.

**Conclusion (s):**

This study presents a preliminary, hypothesis‐generating SD framework for representing potential relationships among organizational factors, individual factors, and nursing work performance. Future studies should validate the model’s generalizability across different healthcare settings and countries and incorporate real‐world implementation outcomes to further evaluate its practical effectiveness.

**Implications for Nursing Management:**

This model identified organizational factors and nursing work performance outcomes that warrant priority testing in future empirical research. Because the model has not been externally calibrated or validated using real‐world longitudinal data, its results should primarily be used to generate research hypotheses rather than directly inform the design of specific management interventions.

## 1. Introduction

In recent years, the growing public demand for health services has brought increased attention to the healthcare industry. The healthcare sector is central to safeguarding people’s health, and nursing operations constitute a critical component characterized by urgency, complexity, and multidisciplinary collaboration, while simultaneously involving high workloads and stringent standards [1, 2]. Nurses not only need to strictly follow established standards to assist in clinical treatment and patient care according to protocols but also communicate closely with patients and their families and coordinate the work of medical teams [3, 4]. These high‐intensity work characteristics impose significant long‐term work pressure on nurses [5]. Studies have revealed that approximately 18% of nurses quit because of the heavy workload [6]. Continuous stress can affect work status and even lead to operational errors [7]. Approximately 15% of inpatients have encountered health risks due to poor nursing, 6% of which have resulted in serious outcomes [8]. These data indicate that nursing work performance directly affects patient safety and the efficiency of the entire healthcare system.

Nursing work performance is defined as the effectiveness with which nurses fulfill the roles and responsibilities directly related to patient care [9]. It is influenced by various factors, and improving the level of nursing work performance has become the focus of attention for medical institutions and scholars. Lasater et al. used cross‐sectional analysis to explore the impact of the nurse–patient ratio on the average hospital length of stay of patients, highlighting its effectiveness in improving the treatment effect of patients and shortening their hospital length of stay [10]. Prasetya et al. analyzed the impacts of gender, work experience, and nursing ability on nursing work performance using chi‐square and Fisher’s exact tests [11]. Adynski et al. qualitatively examined the impacts of workload, support, material resources, and the work environment on nursing work performance through a literature review [12]. Khatatbeh et al. explored the influence of the level of burnout on the quality of nursing services, highlighting the need to reduce the burnout level of nursing staff and implement interventions at the organizational level [13]. In summary, current research on nursing work performance mainly examines two aspects: (1) the types of nursing work performance and its influencing factors and (2) the relationship between nursing work performance and its influencing factors.

However, existing research has not yet established a unified analytical framework that simultaneously integrates performance types, levels of influencing factors, and dynamic mechanisms. The specific research gaps are as follows:

First, the conceptualization and classification of nursing work performance remain fragmented. Previous studies have used indicators, such as adverse event rates, medication errors, hospital‐acquired infections, patient falls, and nursing errors to represent deficiencies in the quality of nursing care. However, these indicators belong to different conceptual levels, and their boundaries and interrelationships have not been clearly explained. For example, reviews by Li et al. and Jun et al. on occupational burnout simultaneously examined multiple outcomes, including patient safety, quality of care, Patient Satisfaction, organizational commitment, and productivity, indicating that nursing work performance is inherently multidimensional. Nevertheless, existing studies have generally focused on only one or a limited number of performance types and have not sufficiently integrated different performance dimensions [14, 15]. Therefore, it is necessary to systematically identify the major types of nursing work performance and further clarify their conceptual boundaries.

Second, existing research has not adequately distinguished between organizational and individual factors. Nurse staffing, the work environment, and leadership support primarily represent organizational factors, whereas work experience, competence, and occupational burnout mainly reflect individual characteristics. Although previous studies have separately demonstrated associations between nurse staffing, the organizational environment, occupational burnout, and nursing work performance, most have focused on a single factor or pathway. Few studies have clearly distinguished organizational factors from individual factors or explained the relationships among organizational factors, individual factors, and nursing work performance within a unified framework. Therefore, an analytical framework is needed that clearly differentiates organizational‐level and individual‐level factors and explains their cross‐level pathways of influence.

Third, existing studies have predominantly used static methods, such as cross‐sectional surveys, correlational analyses, and qualitative reviews, which are limited in their ability to capture dynamic feedback mechanisms within nursing systems. Using a theory‐driven rapid realist review, Rapin et al. examined the contexts and mechanisms through which feedback on nursing‐sensitive indicators operates. Their findings suggested that nursing work performance is not linearly determined by a single factor but is influenced by a complex feedback system comprising the organizational context, feedback mechanisms, the implementation of actions, and other interacting factors. Therefore, greater attention should be paid to temporal processes, feedback pathways, and dynamic evolutionary mechanisms in the improvement of nursing work performance [16].

System dynamics (SD) is a methodology used to simulate and predict the behavior of complex systems. A system refers to “a collection of elements that continually interact over time to form a unified whole.” Dynamics means change over time, where variables and parameters change their values over time. Therefore, SD is a method that is used to study how a system changes over time. Sharif et al. applied SD models to examine how healthcare systems and pharmaceutical supply chains affected patient morbidity and mortality during the COVID‐19 pandemic [17]. Bei et al. integrated game theory with SD to analyze decision‐making behaviors and interaction mechanisms among governments, port enterprises, and shipping companies regarding the adoption of green methanol, providing insightful strategies for the promotion of green methanol‐fueled ships [18]. Farid et al. used SD modeling to quantify and understand the impact of nursing workload on nurse burnout, absenteeism, and the quality of care [19].

Compared with static approaches that primarily examine unidirectional associations, SD can represent feedback relationships among variables through causal loop diagrams and describe the accumulation, inflows, outflows, and time delays of state variables through stock‐and‐flow models. Previous research has indicated that SD can simultaneously address feedback, interactions among variables, time delays, and reciprocal causal relationships, and can be used to assess the potential consequences of long‐term strategic changes [20]. In healthcare research, SD has also been used to simulate interactions among Resource Allocation, patient flows, and policy interventions, thereby supporting managers in comparing the potential system‐level outcomes of different policy scenarios [21, 22]. SD is therefore well suited to addressing the research questions of this study. First, it can integrate different types of nursing work performance within a unified system boundary, avoiding the use of a single outcome variable to represent overall performance. Second, it can distinguish between organizational‐level and individual‐level variables and depict the cross‐level pathways among organizational factors, individual factors, and nursing work performance. Finally, it can incorporate feedback loops, cumulative effects, and time delays into the model to explore potential trends in nursing work performance arising from the combined effects of multiple factors.

In summary, based on the systematic identification and classification of nursing work performance and its organizational‐ and individual‐level influencing factors, this study applies SD to construct a dynamic feedback model linking these three components, with the aim of revealing their pathways of influence and feedback relationships. Scenario simulations are then conducted to explore potential changes in nursing work performance under different adjustments to organizational factors. It should be emphasized that, in the absence of real‐world data, the primary purpose of applying SD in this study is not to make deterministic predictions of future performance, but to explore potential performance trends under different parameter settings and management strategies based on the available evidence.

## 2. Methods

### 2.1. Research Objectives and Framework

We first conducted a literature review to systematically extract various types of nursing work performance and their influencing factors for examining nursing operations. Subsequently, we employed SD to quantitatively explore the relationships among these factors and nursing work performance. The overall research framework is depicted in Figure 1.

**FIGURE 1:**
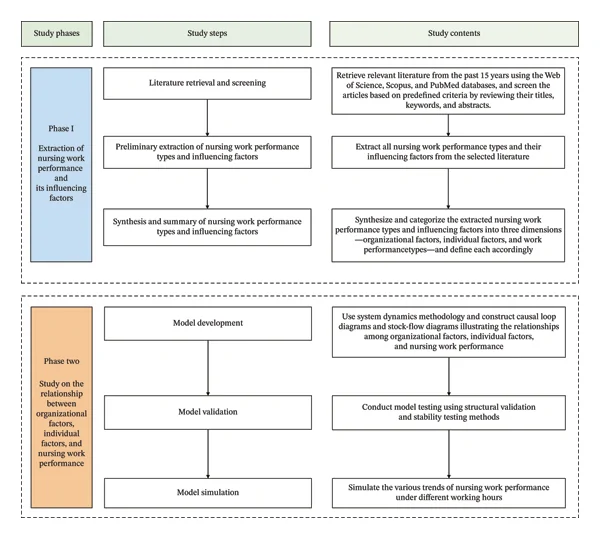
Two‐stage research framework. Note: Stage 1 included the literature search and screening and the extraction and synthesis of nursing work performance and its influencing factors. Stage 2 included the development and validation of the system dynamics model and model simulations under the reference run and alternative implementation‐fraction scenarios.

### 2.2. Literature Review

#### 2.2.1. Literature Search Strategy

The overall search framework and database‐specific search strategies were adapted from our previous review of factors influencing healthcare professionals’ work performance [23]. The factors identified in that review also served as the initial conceptual basis for the present study. The previous review covered publications from 2010 to 2023 and identified 10 microlevel factors and nine macrolevel factors through bibliometric analysis and supplementary comparative analysis. In the present study, we extended the search end date to December 31, 2024, and conducted a new round of literature screening using inclusion and exclusion criteria developed specifically for nursing work performance. We then re‐examined the definitions and conceptual boundaries of the relevant factors and reclassified them into seven organizational factors and three individual factors. We also defined five categories of nursing work performance in the present study.

In this study, the systematic review of the literature related to work performance consisted of two stages: planning and implementation. The literature review protocol was retrospectively registered on the Open Science Framework (OSF) on June 24, 2026. The OSF registration is available at https://osf.io/7r4hv. First, in the planning stage, key elements, such as the time span of the literature review, the databases used, and the search keywords, were clarified. Specifically, the review covered a 15‐year period from January 1, 2010, to December 31, 2024, and the databases covered were PubMed, Scopus, the Web of Science Core Collection, and the ProQuest Nursing & Allied Health Database. The specified literature category pertained to studies published in English, and the keyword combinations used were as follows: (“nurse” OR “health care workers” OR “healthcare workers” OR “health care providers” OR “primary care providers” OR “frontline health workers” OR “care providers” OR “medical workers” OR “medical”) AND (“job performance” OR “employee’s performance” OR “work performance” OR “nursing performance”) AND (factor∗ OR determinant∗ OR predictor∗ OR antecedent∗). Specifically, in PubMed, both MeSH terms and free‐text terms were used. In Scopus, the TITLE‐ABS‐KEY field was used for the search. In the Web of Science Core Collection, the TS field was used. In the ProQuest Nursing & Allied Health Database, the NOFT field, representing “Anywhere except full text,” was used. The detailed database search strategies are presented in Table 1.

**TABLE 1:** Database search strategies.

Databases	Search strategy
Web of Science Core Collection	TS=((“nurse” OR “health care workers” OR “healthcare workers” OR “health care providers” OR “primary care providers” OR “frontline health workers” OR “care providers” OR “medical workers” OR “medical”) AND (“job performance” OR “employee’s performance” OR “work performance” OR “nursing performance”) AND (factor^∗^ OR determinant^∗^ OR predictor^∗^ OR antecedent^∗^))
Scopus	TITLE‐ABS‐KEY((“nurse” OR “health care workers” OR “healthcare workers” OR “health care providers” OR “primary care providers” OR “frontline health workers” OR “care providers” OR “medical workers” OR “medical”) AND (“job performance” OR “employee’s performance” OR “work performance” OR “nursing performance”) AND (factor^∗^ OR determinant^∗^ OR predictor^∗^ OR antecedent^∗^))
PubMed	(“nurse”[Title/Abstract] OR “health care workers”[Title/Abstract] OR “healthcare workers”[Title/Abstract] OR “health care providers”[Title/Abstract] OR “primary care providers”[Title/Abstract] OR “frontline health workers”[Title/Abstract] OR “care providers”[Title/Abstract] OR “medical workers”[Title/Abstract] OR “medical”[Title/Abstract]) AND (“job performance”[Title/Abstract] OR “employee’s performance”[Title/Abstract] OR “work performance”[Title/Abstract] OR “nursing performance”[Title/Abstract]) AND (factor^∗^[Title/Abstract] OR determinant^∗^[Title/Abstract] OR predictor^∗^[Title/Abstract] OR antecedent^∗^[Title/Abstract])
ProQuest Nursing & Allied Health Database	NOFT((“nurse” OR “health care workers” OR “healthcare workers” OR “health care providers” OR “primary care providers” OR “frontline health workers” OR “care providers” OR “medical workers” OR “medical”) AND (“job performance” OR “employee’s performance” OR “work performance” OR “nursing performance”) AND (factor^∗^ OR determinant^∗^ OR predictor^∗^ OR antecedent^∗^))

*Note:* The searches covered English‐language studies on nursing work performance and its determinants published between January 1, 2010, and December 31, 2024. TS denotes the Topic field in the Web of Science Core Collection; TITLE‐ABS‐KEY denotes the title, abstract, and keyword fields in Scopus; MeSH denotes Medical Subject Headings; Title/Abstract denotes the free‐text title, abstract, and author‐keyword fields in PubMed; and NOFT denotes “Anywhere except full text” in the ProQuest Nursing & Allied Health Database.

#### 2.2.2. Inclusion and Exclusion Criteria

The inclusion criteria were as follows: (1) studies published between January 1, 2010, and December 31, 2024; (2) studies involving nurses or other nursing personnel; studies involving mixed healthcare personnel samples were included only when findings relating specifically to nursing personnel could be extracted; (3) studies focusing on performance‐related topics, such as job performance, employee performance, or nursing work performance; and (4) studies reporting organizational or individual factors associated with work performance in the results section.

The exclusion criteria were as follows: (1) studies focusing only on work performance in the healthcare industry rather than the work performance of healthcare personnel; (2) studies that did not clearly define or describe the influencing factors, such as those that only mentioned broad terms, such as “organizational factors” or “work environment” without specifying their dimensions, indicators, or meanings; (3) studies that only listed possible influencing factors but did not explain their effects on work performance through statistical analysis, qualitative evidence, theoretical interpretation, or empirical discussion.

#### 2.2.3. Literature Screening

During the literature screening stage, the literature search was conducted according to the predefined items established in the protocol, and a total of 2289 records were identified. This study did not obtain unpublished research materials through manual reference list tracing, gray literature searches, or contact with experts. Two researchers independently conducted title and abstract screening and full‐text assessment according to the predefined inclusion and exclusion criteria. Before screening, standardized training was provided to ensure a consistent understanding of the inclusion and exclusion criteria. To assess inter‐rater agreement, Cohen’s kappa was calculated based on the independent screening results of the studies that entered the full‐text assessment stage. The kappa value was 0.82, indicating high agreement between the two researchers [24]. Any disagreements during the screening process were first resolved through discussion between the two researchers; if consensus could not be reached, a third researcher was invited to make the final decision. Journal names and author information were not blinded during the screening process. The specific literature screening process was conducted according to the PRISMA flow diagram, as shown in Figure 2.

**FIGURE 2:**
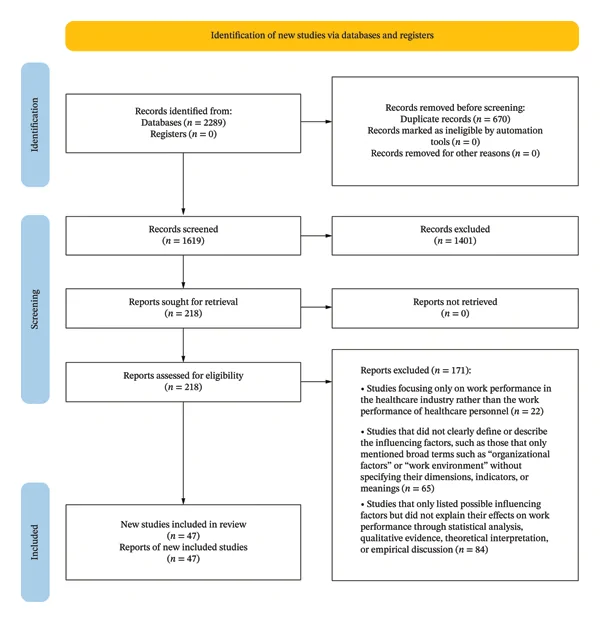
PRISMA 2020 flow diagram for literature screening [25]. Note: A total of 2289 records were identified through the Web of Science Core Collection, Scopus, PubMed, and ProQuest Nursing & Allied Health Database. After 670 duplicate records were removed, the titles and abstracts of 1619 records were screened. The full texts of 218 articles were assessed for eligibility, and 47 studies were ultimately included. PRISMA denotes Preferred Reporting Items for Systematic Reviews and Meta‐Analyses. The completed PRISMA 2020 checklist, including the manuscript page corresponding to each applicable reporting item, is provided in Supporting Information 1.

#### 2.2.4. Data Extraction and Quality Appraisal

During data collection, this study used a standardized data extraction form, which included the basic characteristics of the studies, namely publication year, title, authors, and journal name, as well as nursing work performance, organizational factors, and individual factors. Data were independently extracted by two researchers, and any disagreements were handled using the same procedure as that applied during literature screening. In addition, because this form was primarily used to collect basic study characteristics and relevant findings rather than to develop new measurement constructs or scale instruments, it was not pilot‐tested.

To ensure the validity of the evidence synthesis, this study used the Joanna Briggs Institute (JBI) critical appraisal tools to assess the methodological quality of the 47 included studies. The assessment results showed that 39 studies were of high quality and 8 studies were of moderate quality. Full study‐level JBI appraisal results and the appraisal criteria used in this review are provided in Supporting Information 2.

#### 2.2.5. Narrative Synthesis and Classification

This study adopted a narrative synthesis approach, namely semantic grouping combined with consensus discussion, to identify, summarize, and classify nursing work performance, organizational factors, and individual factors. This process was conducted through group discussions among three researchers. Taking the classification of nursing work performance types as an example, the specific process was as follows:

1. Factors with similar meanings or overlapping definitions were merged. For example, nursing time, blood collection time, and patient transfer time all reflected whether nurses could complete nursing procedures and treatment‐related tasks quickly and in an orderly manner; therefore, they were merged into one category.
2. Contextual distinctions were made for factors that might be ambiguous in the preliminary classification. For example, regarding medication errors, some studies emphasized the impact of medication errors on nursing quality, whereas other studies discussed the need for Process Optimization from the perspective of nursing workflow. Therefore, medication errors were classified into Quality Safety and Process Optimization according to their different attributes.
3. Each category was named. After factor merging and ambiguity resolution were completed, the common characteristics of the factors within each category were further compared, and each category was given a summary name, resulting in the five types of work performance referred to in this study.
4. The classification results were validated. To further examine the rationality and practical applicability of the five types of nursing work performance, this study invited medical safety managers from three healthcare institutions to validate the classification results. The validation mainly focused on whether the five performance types could comprehensively cover the main manifestations of nursing work performance, whether the category names accurately reflected the shared characteristics of the factors within each category, and whether the assignment of each factor to its corresponding category was consistent with understanding in clinical nursing management and medical safety practice. Overall, the medical safety managers considered that this classification framework had good clarity and practical explanatory value and could reflect the main dimensions of nursing work performance in terms of quality and safety, efficiency, and Process Optimization.

Meanwhile, the classification of organizational factors and individual factors was completed using the same procedure.

#### 2.2.6. Evidence Certainty Assessment

Because the included studies showed substantial heterogeneity in study design, variable measurement, statistical methods, and effect‐size reporting, a meta‐analysis was not conducted. Therefore, this study referred to the approach proposed by Murad et al. for rating the certainty of a body of evidence generated from narrative synthesis in the absence of a single pooled estimate of effect. The three pathways in the theoretical framework were treated as separate bodies of evidence: (1) R1, the influence of organizational factors on individual factors; (2) R2, the influence of individual factors on nursing work performance; and (3) R3, the feedback influence of nursing work performance on organizational factors. The certainty of evidence was assessed separately for each pathway [26].

On this basis, two reviewers assessed each body of evidence in terms of five GRADE domains: risk of bias, inconsistency, indirectness, imprecision, and publication bias. Disagreements during the assessment process were resolved with the assistance of a third reviewer. Risk of bias was judged mainly according to study design, measurement methods, use of self‐reported data, control of confounding factors, and risk of selective reporting. Inconsistency was judged according to whether the direction and magnitude of effects were consistent across studies within the same theoretical pathway. Indirectness was judged according to the degree of alignment between the study population, exposure variables, outcome variables, and the corresponding theoretical pathway. Imprecision was assessed according to the number of studies, sample size, reporting of effect estimates, and whether the results were sufficient to support a stable judgment. Publication bias was assessed narratively, mainly based on factors, such as language restrictions and whether gray literature was included. The certainty of evidence for each body of evidence was finally rated as high, moderate, low, or very low. A summary of findings table was developed to present the judgments for the five GRADE domains and the final certainty of evidence for R1, R2, and R3. The complete mapping of the 47 included studies to pathways R1, R2, and R3 is provided in Supporting Information 3.

### 2.3. System Dynamics (SD)

SD is an interdisciplinary subject that studies the behavior and structure of complex systems whose dynamic characteristics can be analyzed from an overall perspective. This method involves the construction of a system structure model using causal relationship, flow diagrams, and quantitative mathematical equations to reveal the internal connection among elements. The healthcare system is a typically complex system, and its performance is influenced by multiple levels and dimensions of factors interacting with each other. Its characteristics include nonlinearity, delayed feedback, and path dependence [1, 3, 4, 6]. This study used the SD method for the expression of the system model because of its advantages in the aforementioned complex systems, namely through the construction of stock‐and‐flow models and feedback loops to precisely depict the complex system’s dynamic change mechanism.

The causal assumptions of this model were mainly based on the Human Factors Analysis and Classification System (HFACS) and the Systems Engineering Initiative for Patient Safety (SEIPS) model [27, 28]. The HFACS model suggests that organizational factors influence nursing work performance by affecting individual factors, such as working conditions, psychological status, fatigue level, ability utilization, and behavioral performance [27]. The SEIPS model also indicates that system factors, including organization, tasks, tools and technologies, and environment, affect individuals’ cognitive load, work stress, professional ability utilization, and task execution processes, and ultimately influence outcomes, such as safety, quality, efficiency, and performance [28]. Therefore, when analyzing the effects of organizational factors on nursing work performance, individual factors represent an important mediating link that cannot be ignored. At the same time, with the development of artificial intelligence technologies and the continuous upgrading of medical equipment, the automation, intelligence, and standardization of some nursing tasks have continued to improve. This may, to some extent, reduce reliance on individual experience and immediate judgment, thereby weakening the influence of some individual factors and helping to improve Rescue Efficiency.

SD modeling mainly comprises three stages: model development, model testing, and model simulation [16]. The model construction of this study focuses on these three stages.

The present study is methodologically related to our previous SD study of nursing workload and medication errors [1]. Both studies follow the general SD research process; however, they differ fundamentally in their subject matter, data, objectives, and findings.

### 2.4. G1 Method and Ridge Regression Analysis

Order relation analysis (G1) is based on the Analytic Hierarchy Process (AHP) and addresses the AHP method’s failure, thereby meeting the uniformity requirement when applied to complex problems. Currently, methods, such as the AHP and Fuzzy Analytic Hierarchy Process (FAHP), are used for subjective weight assignment in research. The implementation process of the G1 method in this study is as follows: First, experts ranked the independent variable indicators from high to low based on their impact on the dependent variable. Second, researchers compared adjacent indicators pairwise and assigned importance scores (1.0 = “equally important,” 1.2 = “slightly important,” 1.4 = “obviously important,” 1.6 = “strongly important,” and 1.8 = “extremely strongly important”; scores of 1.1, 1.3, 1.5, and 1.7 could also be used to indicate intermediate levels of importance between the above categories). Third, researchers summarized and calculated the weights of each independent variable indicator according to the preset mathematical formula.

Using the Psychological State equation as an example, a worked calculation is provided to demonstrate the parameterization process, including the initial ranking, adjacent‐factor importance comparisons, calculation of the final weights, and derivation of the resulting equation. Psychological State is denoted as *P*3, and the seven organizational factors are denoted as *O*1 (Cultural Climate), *O*2 (Leader Attitude), *O*3 (Supervisory Governance), *O*4 (Incentives and Support), *O*5 (Resource Allocation), *O*6 (Task Allocation), and *O*7 (Education and Training). First, individual weights were calculated based on the ranking and adjacent‐factor comparisons provided by each expert. The arithmetic means of the individual weights across all experts were then calculated to obtain the final weights used in the model.

#### 2.4.1. Initial Ranking and Adjacent‐Factor Importance Comparisons

Anonymous expert E01 ranked the seven organizational factors according to the magnitude of their influence on Psychological State and compared the relative importance of each pair of adjacent factors in the resulting order. The ranking from highest to lowest is as follows:

(1) O1>O4>O2>O5>O7>O3>O6.

According to the resulting order, the weights were sequentially denoted as *w*_1_, *w*_2_, *w*_3_, *w*_4_, *w*_5_, *w*_6_, and *w*_7_. The adjacent‐factor comparison value is defined as follows:

(2) rk=wk−1/wk, k=237,,…,.

Based on the adjacent‐factor comparisons above:

(3) r2=1.31.31.21.1,r3=,r4=,r5=r6=r7=.

#### 2.4.2. Calculation of the Individual G1 Weights

According to the G1 method, the weight of the last‐ranked factor was calculated first, after which the remaining weights were derived recursively in reverse order using the adjacent‐factor comparison values. The weight of the last‐ranked factor is calculated as follows:

(4) wm=1+∑k=2m∏i=kmri−1.

After substituting the six adjacent‐factor comparison values, the weight of Task Allocation, which was ranked seventh, is calculated as follows:

(5) w7=0.090795.

The remaining weights were then derived sequentially using the following recursive equation:

(6) wk−1=rkwk, k=762,,…,,w6=1.10.0907950.099874×=,w5=1.10.0998740.109862×=,w4=1.10.1098620.120848×=,w3=1.20.1208480.145018×=,w2=1.30.1450180.188523×=,w1=1.30.1885230.245080×=.

#### 2.4.3. Expert‐Group Mean Weights and Final Equation

The individual G1 weights of each expert were calculated using the procedure described above. For the *j*th organizational factor, the expert‐group mean weight is calculated as follows:

(7) w¯j=1N∑i=1Nwij,

where *w*_*i* *j*_ represents the individual weight assigned to the *j*th organizational factor by the *i*th expert, and *N* represents the number of experts included in the calculation. After the arithmetic means of the individual weights across all experts were calculated and rounded to three decimal places, the mean weights were 0.160 for *O*1, 0.192 for *O*2, 0.105 for *O*3, 0.192 for *O*4, 0.077 for *O*5, 0.132 for *O*6, and 0.142 for *O*7. The seven mean weights summed to 1.000.

Substituting the expert‐group mean weights into the Psychological State equation yielded

(8) P3=0.1600.1920.1050.1920.0770.1320.142O1+O2+O3+O4+O5+O6+O7.

That is, Psychological State = 0.160 × Cultural Climate + 0.192 × Leader Attitude + 0.105 × Supervisory Governance + 0.192 × Incentives and Support + 0.077 × Resource Allocation + 0.132 × Task Allocation + 0.142 × Education and Training. Thus, the experts’ original rankings and adjacent‐factor comparisons were transformed, through the calculation of individual G1 weights and aggregation of the expert‐group means, into the parameters of the equation describing the effects of organizational factors on Psychological State in the SD model.

Ridge regression (also known as L2 regularization) is a biased estimation regression method designed to address severe multicollinearity among independent variables. Variance inflation factor (VIF) is a statistical measure used in regression analysis to detect and quantify multicollinearity. A VIF value greater than 5 indicates significant multicollinearity [29, 30]. In the feedback equations of this study, nursing work performance indicators were set as independent variables, whereas the accumulation rates of organizational factors were set as dependent variables. During the parameterization of the feedback relationships, the nursing work performance indicators associated with each organizational factor were specified as independent variables, whereas the accumulation rate of each organizational factor was specified as the dependent variable. The VIF values for the Cultural Climate, Leader Attitude, Supervisory Governance, Incentives and Support, Resource Allocation, Task Allocation, and Education and Training accumulation‐rate equations were 5.082, 3.132–8.653, 3.277–9.339, 3.192–9.059, 3.219–4.420, 4.087–9.902, and 3.132–8.653, respectively. Multiple VIF values were obtained for a given organizational factor because its regression equation contained multiple independent variables, each with its own VIF; therefore, these values are reported as minimum–maximum ranges. These results indicate varying degrees of multicollinearity among the nursing work performance indicators. Because multicollinearity may lead to numerical instability in ordinary least squares coefficient estimates, this study used ridge regression as an exploratory regularization method.

### 2.5. Expert Interviews

This study adopted a 1‐to‐1 online interview approach to obtain data support. The selection process for experts at each stage is as follows:

#### 2.5.1. Model‐Development Stage

Number of experts: 20.

Discipline: Nursing.

Selection criteria: More than 5 years of clinical work experience.

#### 2.5.2. Model Testing Stage

Number of experts: 4.

Discipline: Nursing, Medical Safety Researcher.

Selection Criteria: Head nurses with more than 20 years of nursing experience (2 people); scholars with more than 10 years of medical safety research experience (2 people).

Specifically, the two rounds of expert interviews were conducted for model development and model validation, respectively. Their procedures are described below in terms of the interview process, interview content, data recording, and the handling of expert opinions.

Model‐Development Stage:

1. Interview Process: Twenty nursing professionals with more than five years of clinical experience were recruited for individual online interviews lasting approximately 60–90 min. Before each interview, the researcher explained the study purpose, interview content, intended use of the data, and confidentiality principles to the expert. After obtaining informed consent, the researcher introduced the variable definitions and evaluation criteria in a standardized sequence and then conducted the scoring, ranking, and adjacent‐factor importance comparison tasks in the same order. After the interview, the researcher cross‐checked the electronic records against the audio‐recording to ensure consistency.
2. Interview Content: The interview comprised three main components. First, experts rated the lower‐ and higher‐state levels of the individual factors on a scale of 1–100 and subsequently rated the levels of nursing work performance under these two individual‐factor states. These data were used to parameterize the relational equations. Second, experts rated, on a scale of 1–100, the degree to which each organizational factor would need to be improved under the two nursing work performance scenarios described above. A higher score indicated that a greater adjustment to the corresponding organizational factor would be required. Ridge regression was then performed using the nursing work performance levels as the independent variables and the scores for the required degree of improvement in the organizational factors as the dependent variables to specifying the hypothesized feedback relationships of nursing work performance on organizational factors. Third, following the G1 method, experts evaluated the relative influence of the relevant independent variables on each dependent variable. Specifically, they first ranked the factors from highest to lowest according to their degree of influence and then compared the importance of adjacent factors. The relative weight of each factor was calculated from these results.
3. Data Recording: The researcher used a standardized structured questionnaire to record each expert’s ratings, rankings, and adjacent‐factor comparison values item by item. The interviews were also audio‐recorded, primarily to verify the consistency of the information recorded in the structured questionnaires.
4. Handling of Expert Opinions: Each expert’s independent judgments were retained, and their individual ratings and weights were compiled and calculated separately. The final results used to parameterize the SD model were then obtained by calculating the mean weights across the expert group. Thus, differences among expert judgments were preserved, while the evaluations of different experts were synthesized using the expert‐group mean weights.

Model Validation Stage:

1. Interview Process: A separate, nonoverlapping panel of four model evaluators was recruited, comprising two nurse managers with approximately 20 years of nursing experience and two university scholars with more than 10 years of experience in healthcare safety research. None of the four evaluators participated in the model‐development interviews, ratings, rankings, G1 weight elicitation, or ridge regression parameterization conducted with the 20 model‐development experts. Individual online interviews lasting approximately 60–90 min were conducted. Before each interview, the researcher explained the study purpose, validation content, intended use of the data, and confidentiality principles to the expert. After obtaining informed consent, the researcher provided standardized explanations of the model validation dimensions and assessment criteria. During the formal interview, the causal loop diagram, stock‐and‐flow diagram, and preliminary simulation results were presented to each expert in the same sequence. The researcher also explained the model variables, system boundaries, causal relationships, feedback loops, and key parameter settings. The experts were then asked to evaluate the model item by item according to the predefined validation dimensions and to propose revisions. After the interview, the researcher cross‐checked the validation records against the audio‐recording to ensure consistency.
2. Interview Content: Before the evaluation interviews began, we developed a structured evaluation form and prespecified the following qualitative evaluation criteria: (1) the practical relevance of the model variables; (2) the appropriateness of the stocks, flows, and auxiliary variables; (3) the plausibility of the directions and logic of the causal relationships; (4) the closure of the main feedback loops; (5) the appropriateness of the system boundary; (6) the coverage of major factors within the defined boundary; and (7) the qualitative plausibility and consistency of the direction of model behavior under parameter perturbations.
3. Data Recording: The researcher used a standardized structured questionnaire to record each expert’s judgments, rationales, and proposed revisions regarding the completeness of the model structure, the rationality of the causal relationships, the closure of the feedback loops, the appropriateness of the system boundaries, and operational stability. The interviews were also audio‐recorded, primarily to verify the consistency of the information recorded in the structured questionnaires.
4. Handling of Expert Opinions: Each expert’s independent judgments regarding the validation items were retained, and the evaluative comments and proposed revisions were compiled. The researchers reviewed each expert opinion in relation to the relevant literature, model structure, and simulation results. Following expert discussion and review, the experts concluded that the model variables, causal relationships, feedback loops, system boundaries, and key parameter settings were generally reasonable.

The expert‐interview and evaluation protocol, including audio‐recording and the classification‐review and model‐evaluation activities, received prospective approval from the Ethics Review Committee of Northeastern University. Written informed consent, including consent to audio‐recording, was obtained from all participants before the interviews.

This study used G∗Power 3.1.9.7 to conduct a sensitivity power analysis, with the analysis type set as F tests: Linear Multiple Regression: Fixed model, *R* ^2^ deviation from zero. Because the ridge regression equations used to estimate feedback accumulation rates in this study included 2 to 5 predictors, the most complex regression equation was used as a conservative scenario for the power analysis, with the number of predictors set to 5. Regarding parameter settings, *α* = 0.05 and power = 0.80 were used as the baseline settings for the sensitivity power analysis. This setting was based on Cohen’s statistical power analysis framework [31], which provides the classical definition of the relationships among significance level, effect size, sample size, and statistical power, and is consistent with commonly used power analysis practices in behavioral science, social science, and health research. The results showed that, under these conditions, the overall effect size detectable in this study was *f* ^2^ = 0.94, corresponding to an *R* ^2^ of approximately 0.48. The critical F value was 2.96, with df1 = 5 and df2 = 14. This result indicates that the available sample could detect only a very large overall regression effect and was insufficient to support reliable estimation of small or moderate relationships. Consequently, the ridge regression coefficients derived from 20 experts may have substantial statistical uncertainty and be sensitive to sample composition, limiting the reliable identification of small‐to‐moderate relationships and the precision and generalizability of the simulation results.

### 2.6. Model Indices and Additional Verification

For dimensional consistency testing, the organizational‐factor stocks, individual factor indices, nursing work performance indices, and required improvement scores were all defined as dimensionless variables on the model’s native 0–100 index scale. Organizational‐factor accumulation rates were expressed in index points per month. Required improvement scores were converted into accumulation rates using a dimensionless implementation fraction and an assumed implementation period of 1 month. An initial stock value of 0 indicates that no improvement in organizational factors has accumulated relative to the reference point at the start of the simulation.

For normalization, no empirical min–max normalization or z‐score standardization was applied to the variables. G1 weights were normalized only within their respective equations so that the weights in each equation summed to 1.

For plausible‐bound testing, the assumed plausible ranges were 0–100 for organizational‐factor stocks and derived indices, and 0–1 for implementation fraction and performance gain coefficients. Boundary behavior testing included the 12‐month reference run, 32 management scenarios, and a reference run extended to 120 months.

For the uncertainty analysis, two sets of 5000 joint parameter simulations were conducted, with perturbation ranges of ±10% and ±20% relative to each parameter’s nominal value, respectively. The jointly perturbed parameters included regression intercepts and nonzero feedback slopes, G1 weights, performance gain coefficients, implementation fraction, and implementation periods. After each draw, the G1 weights were renormalized within their respective equations to ensure that their sum remained 1.

The reproducible Vensim model file, including the executable equations, unit annotations, initial values, parameter settings, and simulation controls, is provided in Supporting Information 4. The full textual listing of model equations, initial conditions, and simulation control parameters is available in Supporting Information 5.

## 3. Result

### 3.1. Characteristics of Included Literature

The characteristics of the 47 included studies in terms of geographical distribution, research environment, methodology, and sample size are as follows: The publication years of the studies range from 2010 to 2024, covering nearly 15 years of research. The geographical distribution of the studies included China, the United States, Turkey, Iran, Norway, South Korea, Israel, Spain, Singapore, Argentina, Pakistan, Ethiopia, Oman, Egypt, Jordan, and several other countries and regions. The study settings mainly involved healthcare delivery contexts, including general hospitals, specialized hospitals, outpatient and community health centers, and specialized clinical units, such as emergency departments and operating rooms. Questionnaire surveys remained the predominant research method, while focus‐group interviews, semistructured interviews, and retrospective cohort designs were also represented. Sample sizes ranged from 18 to 2946 participants. Overall, these characteristics indicate that the included studies are diverse and representative, providing a foundation for the extraction, classification, and SD modeling of the types of nursing work performance and its organizational and individual factors.

### 3.2. Evidence Certainty Assessment Results

A summary of findings table was developed to present the judgments for the five GRADE domains and the final certainty of evidence for the three bodies of evidence, namely R1, R2, and R3, as shown in Table 2.

**TABLE 2:** GRADE assessment of the certainty of evidence for the three relationships in the system dynamics model.

Evidence	Risk of bias	Inconsistency	Indirectness	Imprecision	Publication bias	Final certainty rating
R1: Influence of organizational factors on individual factors	Some risk of bias	Not serious	Not serious	Not serious	Potential risk	Moderate certainty
R2: Influence of individual factors on nursing work performance	Some risk of bias	Not serious	Not serious	Not serious	Potential risk	Moderate certainty
R3: Influence of nursing work performance on organizational factors	Some risk of bias	No obvious inconsistency, but the judgment was limited	Some indirectness	Some imprecision	Potential risk	Low certainty

*Note:* R1 denotes the influence of organizational factors on individual factors; R2 denotes the influence of individual factors on nursing work performance; and R3 denotes the feedback influence of nursing work performance on organizational factors. The assessment domains included risk of bias, inconsistency, indirectness, imprecision, and publication bias.

The GRADE evidence certainty assessment showed that the influence of organizational factors on individual factors and the influence of individual factors on nursing work performance were both rated as having moderate certainty. Although these two bodies of evidence were limited by observational study designs, self‐reported measurements, insufficient control of confounding factors, and potential publication bias, the number of relevant studies was relatively large, the overall direction of the findings was generally consistent, and the theoretical mechanisms were relatively clear. In contrast, the feedback influence of nursing work performance on organizational factors was rated as having low certainty, because this relationship was mainly supported by management feedback logic and SD theory, while direct empirical evidence was relatively limited [32, 33].

R1 represents the influence of organizational factors on individual factors and was supported by 25 studies. The included studies predominantly used cross‐sectional or observational designs, and some relied on self‐reported data without adequately controlling for confounding factors; therefore, this body of evidence had certain limitations regarding risk of bias. Most studies reported consistent directions of association, resulting in a favorable assessment of inconsistency. The study populations, organizational factors, and individual factors generally corresponded to the variables in our model, although a small number of studies involved mixed groups of healthcare professionals or specific healthcare settings, resulting in minor concerns regarding indirectness. Some studies did not fully report effect sizes and their confidence intervals, resulting in certain concerns regarding imprecision. Because only English‐language publications were included and gray literature was not systematically searched, a potential risk of publication bias remained. Considering the number of studies, the consistency of the findings, and the available theoretical and empirical support, the certainty of evidence for R1 was rated as moderate.

R2 represents the influence of individual factors on nursing work performance and was supported by 36 studies. The included studies were also predominantly cross‐sectional or observational, and some used self‐reported measures without adequately controlling for confounding factors; therefore, this body of evidence had certain limitations regarding risk of bias. The directions of the associations reported across the studies were generally consistent, resulting in a favorable assessment of inconsistency. Most studies directly examined nurses, although some involved other healthcare professionals, resulting in certain concerns regarding indirectness. Some studies did not fully report effect sizes and their confidence intervals, resulting in certain concerns regarding imprecision. Publication bias was also possible and therefore remained a potential concern. Considering the relatively large number of studies, the consistency in the direction of the findings, and the clear theoretical and empirical basis, the certainty of evidence for R2 was rated as moderate.

R3 represents the feedback influence of nursing work performance on organizational factors and was supported by only two relevant studies. The limited number of studies and inadequate control of confounding factors in some studies resulted in substantial concerns regarding risk of bias. Differences in the performance indicators, organizational factors, and healthcare settings examined made it difficult to reach a stable judgment regarding inconsistency. The relevant studies primarily focused on the effects of organizational management measures on nursing work performance and therefore provided only indirect support for the feedback relationship, resulting in substantial concerns regarding indirectness. The available studies did not report the magnitude of the feedback effects or their confidence intervals, resulting in substantial concerns regarding imprecision. Publication bias was also possible and therefore remained a potential concern. Considering these factors, the certainty of evidence for R3 was rated as low.

The low certainty of R3 means that the feedback relationship between nursing work performance and organizational factors cannot yet be regarded as a fully validated causal mechanism. This feedback pathway is primarily intended to describe a potential process of organizational learning and managerial adjustment. For example, when Rescue Efficiency or Quality Safety declines, managers may further adjust Task Allocation, Education and Training, Supervisory Governance, or communication mechanisms. Although this logic is theoretically grounded, sufficient empirical evidence is currently lacking to determine its actual direction, magnitude, and time delay. Therefore, the feedback loops in the model are primarily used to explore potential dynamic mechanisms and generate research hypotheses. The corresponding simulation results should be interpreted as conditional outcomes under the specified model structure and parameter assumptions.

### 3.3. Types of Nursing Work Performance and Their Influencing Factors

Through a literature review, this study summarized the types of nursing work performance and their influencing factors. Based on the three‐dimensional model of healthcare quality proposed by Donabedian [34], the influencing factors were categorized into three levels—performance, individual, and organizational levels—according to the “outcome‐process‐structure” framework.

#### 3.3.1. Types of Nursing Work Performance

In Donabedian’s three‐dimensional model, outcomes are regarded as the ultimate verification of quality. In the nursing field, work performance, as an outcome of nursing practice, has attracted much attention. Despite extensive prior studies on nursing work performance, the types of nursing work performance suffer from problems of repetition, intersection, or unclear meaning boundaries. This section summarizes the types of nursing work performance extracted from 47 studies in the literature review from the two aspects of unified term expression and clarifying meaning boundaries. With regard to the former, previous studies on nursing work performance have involved nursing time, blood collection time, patient transfer time, and so on. Considering that they all reflect the behavioral evaluation of nursing staff’s rapid and orderly nursing operations and treatments for patients, this study categorizes them as “Rescue Efficiency.” With regard to the latter, studies pertaining to medication errors have investigated the quality of nursing or the reliability of the nursing work process. Considering that the same event is investigated for different reasons, this study respectively classifies them as “quality safety” and “process optimization.” Following this process, the 21 terms identified in the included studies were consolidated into five performance‐related constructs: Quality Safety, Rescue Efficiency, Patient Satisfaction, Job Satisfaction, and Process Optimization. Their definitions are presented in Table 3. To further clarify their conceptual organization, we drew on the SEIPS model, which proposes that the components of a healthcare work system influence work and clinical care processes, which in turn affect patient, employee, and organizational outcomes [28]. Guided by this framework, the five constructs were organized within a broader nursing‐system performance framework into three conceptual domains: (1) care‐process and operational performance, comprising Rescue Efficiency and Process Optimization; (2) patient‐related outcomes, comprising Quality Safety and Patient Satisfaction; and (3) nurse‐related outcomes, represented by Job Satisfaction.

**TABLE 3:** Five types of nursing work performance and their definitions.

Performance	Definitions
Quality safety	Level of medical care quality that nurses provide to patients, including adherence to clinical guidelines, administration of correct treatments, and achievement of optimal patient health outcomes.
Rescue efficiency	Extent to which nursing staff—supported by departmental and equipment resources—carry out rapid, orderly care and interventions to ensure patients receive necessary medical services in a timely manner.
Patient satisfaction	Degree to which patients and their families are satisfied with nurses’ professional skills, communication style, emotional support, and overall care experience.
Job satisfaction	Level of nurses’ satisfaction with their work environment, job content, and career development, as reflected in measures, such as Job Satisfaction, turnover intention, and staff turnover rates.
Process optimization	Effectiveness of corrective measures proposed by nursing staff to address issues in care processes (e.g., prolonged transfer times) and adverse events (e.g., infusion extravasation and medication errors), as well as improvements to care plans (e.g., targeted medication adjustments to accelerate recovery).

*Note:* The five types of nursing work performance and their definitions were derived from the 47 studies included in this review.

#### 3.3.2. Influencing Factors of Nursing Work Performance

In Donabedian’s three‐dimensional model, structural assessment covers macrolevel factors, such as facilities, staff qualifications, and management systems, while process assessment focuses more on microlevel factors during work processes. Based on Donabedian’s three‐dimensional model, combined with the Social Cognitive Theory (SCT) and the influencing factors of medical error behaviors proposed by Jin et al. [34, 35], this study systematically categorizes the influencing factors of nursing work performance extracted from the literature into two aspects: organizational factors and individual factors. The specific process of summarizing organizational factors is as follows:

First, the term expression is unified by identifying and merging repeated or highly similar semantic contents in previous studies. For example, the descriptions of human resources, strategies, shifts, and workload on the Task Allocation and human resource configuration of nursing staff are categorized as “Task Allocation.”

Second, factors or definitions not clearly mentioned in previous studies are closely examined. For example, with regard to nursing staff skills training, existing studies primarily examine practical ability, paying attention to the assessment of theoretical knowledge and the practical skills of nursing staff. Because they both require organizational‐level planning, this study expands the concept of training to include theoretical knowledge education content, classifying such factors under “Education and Training.”

Finally, this study clarifies the meaning in previous studies. For example, in terms of management factors, some studies focus on the Leader Attitude, while others focus on their behaviors in daily work. Considering that different perspectives focus on the same object, this study classifies these factors as “Leader Attitude” and “Supervisory Governance,” respectively.

Following the above method, the 19 factors extracted from previous studies were summarized, resulting in seven organizational factors (including Cultural Climate, Leader Attitude, Supervisory Governance, Incentives and Support, Resource Allocation, Task Allocation, and Education and Training) and three individual factors (including Physiological State, Professional Competence, and Psychological State). The specific types and definitions of organizational factors and individual factors are presented in Tables 4 and 5 below, respectively.

**TABLE 4:** Seven organizational factors and their definitions.

Organizational factors	Definitions
Cultural climate	Long‐standing values, norms, cultural atmosphere, and interpersonal relationships within the nursing department that collectively shape the work ethos and environment.
Leader attitude	Behavioral patterns, communication styles, and attitudes of department leaders during management, guidance, and decision‐making processes toward nursing staff.
Supervisory governance	Activities of overseeing and managing nurses’ daily work, service quality, and professional conduct within the medical institution.
Incentives and support	Various reward and support measures implemented by the medical institution, such as financial incentives and personnel promotions.
Resource allocation	Distribution of material and technical resources by the medical institution, including medical treatment equipment, protective supplies, and disinfectants.
Task allocation	Assignment of nursing tasks and workloads according to departmental operational needs, including shift scheduling and patient distribution.
Education and training	Educational and practical activities organized by the medical institution to enhance nurses’ professional knowledge and skill levels.

*Note:* The seven organizational factors and their definitions were derived from the 47 studies included in this review and represent organizational‐level factors associated with nursing work performance.

**TABLE 5:** Three individual factors and their definitions.

Individual factors	Definitions
Physiological state	Nurses’ physical health and functional status, including sleep quality, health conditions (e.g., musculoskeletal issues, colds, and fevers), and physical fatigue.
Professional competence	Nurses’ levels of professional knowledge, skills, and experience—including theoretical knowledge and technical abilities acquired through education and practice—and their clinical application.
Psychological state	Nurses’ emotional and perceptual status, including burnout, stress levels, and mood.

*Note:* The three individual factors and their definitions were derived from the 47 studies included in this review and represent individual‐level factors linking organizational factors to nursing work performance.

### 3.4. Relationship Between Nursing Work Performance and Its Influencing Factors

This section uses the SD method to construct a model that explores the relationship among organizational factors, individual factors, and nursing work performance.

#### 3.4.1. Definition of System Boundaries and Model Assumptions

In constructing the SD model of nursing work performance and its influencing factors, the precise definition of system boundaries is essential. The definitions of system boundaries and model assumptions in this study mainly include the following aspects. The spatial boundary is limited to the level of a single nursing department, and factors at the cross‐departmental or hospital‐wide level are treated as exogenous inputs. The time scale mainly focuses on the medium term, with the time step set by month to capture dynamic changes, such as Task Allocation and shift scheduling. Model variables are aggregated based on literature induction, retaining seven categories of organizational factors, three categories of individual factors, and five types of nursing work performance. Variables with cumulative properties at the organizational level are used as state variables, and the relevant change rates are used as flow variables. The model assumes that organizational factors act on nursing work performance indirectly by influencing individual factors, while performance levels act on organizational factors through a feedback mechanism, forming a closed loop. The relationships within the loop can be positive or negative, and the model allows for the existence of saturation effects, threshold effects, and explicit time lags (e.g., delays in the effectiveness of training and the implementation of Process Optimization measures). All variables are expressed by quantifiable indicators and adopt a unified dimension. Organizational‐level elements, such as Incentives and Support, Supervisory Governance, and Education and Training, are used as adjustable policy parameters.

#### 3.4.2. Model Development

This study considers organizational factors, individual factors, and types of nursing work performance as variables and incorporates them into the system to conduct SD modeling. Given that organizational factors affect individual factors and individual factors affect nursing work performance, the level of nursing work performance will in turn affect organizational factors through a feedback mechanism, thereby closing the loop, as shown in Figure 3 below.

**FIGURE 3:**
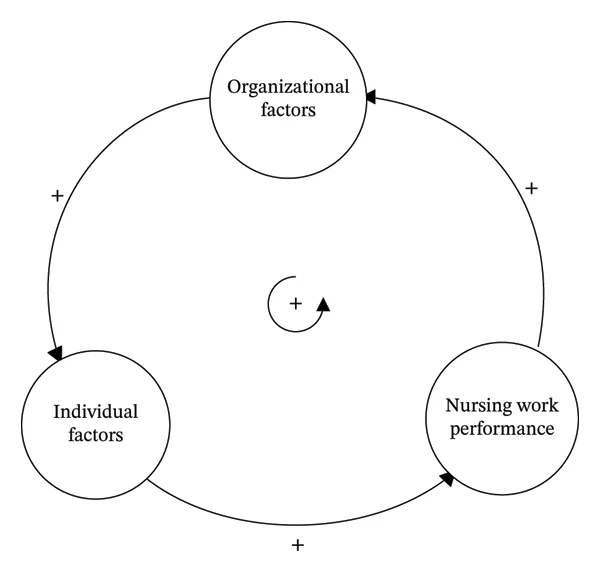
Reinforcing feedback loop among organizational factors, individual factors, and nursing work performance. Note: The arrows indicate the assumed directions of influence between variables. The “+” signs beside the arrows indicate that the two connected variables change in the same direction. The “+” sign in the center indicates a reinforcing feedback loop comprising “organizational factors–individual factors–nursing work performance–organizational factors.”

Figure 3 illustrates the reinforcing feedback relationships among organizational factors, individual factors, and nursing work performance within this study’s system. Specifically, positive organizational factors can promote the positive development of individual factors, and the improvement of individual factors further enhances nursing work performance. The + symbol in Figure 3 indicates positive correlation. Meanwhile, the improvement of performance in turn exerts a positive impact on organizational factors, thereby forming a self‐reinforcing feedback loop at the system level. The + symbol in the center of Figure 3 represents a reinforcing feedback loop.

Based on the aforementioned causal loop diagram, after fully considering the relationships among different variables with the expert team, the following conclusions were drawn. First, in identifying the causes of individual factors, Physiological State is influenced by three factors, namely, Resource Allocation, Task Allocation, and Education and Training; Professional Competence is affected by three factors, namely, Education and Training, Incentives and Support, and Task Allocation; and Psychological State is impacted by seven organizational factors, including Resource Allocation, Task Allocation, Education and Training, and Cultural Climate. All five types of nursing work performance are influenced by the three individual factors. In analyzing feedback loops, the level of Patient Satisfaction affected the variation of seven organizational factors (e.g., Task Allocation and Resource Allocation); Rescue Efficiency impacted six organizational factors (e.g., Task Allocation and Education and Training); and Process Optimization and Job Satisfaction each influenced three organizational factors. Second, in this study, organizational factors were designated as stocks, the variation of organizational factors under the influence of nursing work performance as flows, and both individual factors and types of nursing work performance as auxiliary variables. The SD stock‐and‐flow diagram was drawn using Vensim PLE 10.3.1 software, as shown in Figure 4 below.

**FIGURE 4:**
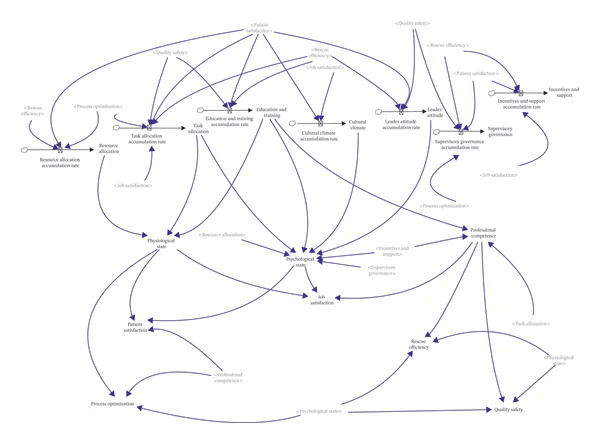
Stock‐and‐flow structure of the system dynamics model of nursing work performance. Note: The seven organizational factors were specified as stock variables, and their accumulation rates were specified as flow variables. The three individual factors and five types of nursing work performance were specified as auxiliary variables. The arrows indicate the directions of influence between variables, and variables enclosed in angle brackets are shadow variables that reference existing variables. The model was developed using Vensim PLE 10.3.1.

Figure 4 presents the SD stock‐and‐flow diagram constructed in this study. The following explanations are provided for it:

1. In terms of the overall structure, Figure 4 consists of three levels: organizational factors, individual factors, and nursing work performance. The organizational‐factors level includes Leader Attitude, Education and Training, Cultural Climate, and other elements that directly affect factors at the individual factors level. The individual factors level covers three individual‐related variables: Psychological State, Professional Competence, and Physiological State. These variables are not only influenced by multiple factors at the organizational‐factors level, but also directly affect the nursing work performance level. The nursing work performance level is mainly reflected in indicators, such as Rescue Efficiency, Quality Safety, and Job Satisfaction. These performance indicators reflect the operational results of the system and influence variables at the organizational‐factors level through feedback effects.
2. The causal relationships between different levels are represented by arrows, with the arrows pointing from the independent variable to the dependent variable. Figure 4 shows multiple causal chains interacting with each other, forming a dynamic feedback mechanism. Within this system, multiple variables interact to collectively form reinforcing feedback loops. For example, a positive Leader Attitude (an organizational factor) can significantly improve the subordinates’ Psychological State (an individual factor); when Psychological State is enhanced, nurses’ Job Satisfaction (one dimension of nursing work performance) also increases. The improvement of Job Satisfaction further strengthens the management’s Leader Attitude, fostering a more positive atmosphere within the organization. Thus, a reinforcing feedback loop of Leader Attitude–Psychological State–Job Satisfaction–Leader Attitude is formed, driving the system into a virtuous cycle.

Based on the system stock‐and‐flow diagram, this study clarified the relationships between different factors and nursing work performance. The following two parts were combined with 1‐to‐1 expert interviews, providing data support for the development of the SD model. First, the G1 method was used to obtain the relative weights of each variable. Second, ridge regression was applied to derive the relationship between the improvement degree of organizational factors and nursing work performance.

The data obtained from the interviews were used for G1 method analysis and ridge regression analysis.

1. For G1 method data analysis, using the model development of Psychological State as an example, Psychological State was regarded as the dependent variable and seven organizational factors (e.g., Cultural Climate, Leader Attitude, and Supervisory Governance) as independent variables. The calculated weights of the independent variables, retained to three decimal places, were 0.16, 0.192, 0.105, 0.192, 0.077, 0.132, and 0.142, respectively. Therefore, the formula for Psychological State is as follows:
(9) Psychological State=0.160.1920.1050.1920.0770.1320.142×Cultural Climate+×Leader Attitude+×Supervisory Governance+×Incentives and Support+×Resource Allocation+×Task Allocation+×Education and Training.
 From the above formula, it can be seen that the Psychological State of employees is most significantly influenced by Leader Attitude and Incentives and Support, followed by Cultural Climate, Education and Training, Task Allocation, etc. Therefore, when hospitals aim to improve the Psychological State of their employees, they should give priority to the factors that have a greater impact. Based on this approach, the research results can provide ideas and basis for improving performance in medical practice.
2. For ridge regression analysis, the acquisition of the feedback relationship between the improvement degree of Task Allocation and the level of nursing work performance was taken as an example. First, the interview data on Quality Safety, Rescue Efficiency, Process Optimization, Patient Satisfaction, and Job Satisfaction were input as independent variables and the data on the improvement degree of Task Allocation as the dependent variable. The ridge parameter was then determined using the ridge trace plot. The ridge parameter was selected according to the principle of minimizing estimation bias while reducing the influence of multicollinearity. Specifically, a relatively small ridge parameter was selected at the point where the signs, relative magnitudes, and trajectories of the standardized regression coefficients began to stabilize. Using the Task Allocation (*O*6) feedback equation as an example, a candidate ridge parameter of *k* = 0.138 was first automatically determined using the VIF method and was subsequently examined using the ridge trace plot. As shown in Figure 5, the standardized regression coefficients changed considerably during the initial increase in *k.* When *k* approached 0.138, the coefficient curves corresponding to Quality Safety, Rescue Efficiency, Process Optimization, Patient Satisfaction, and Job Satisfaction gradually flattened. Therefore, *k* = 0.138 was selected as the ridge parameter for this regression equation.

The resulting regression equation for the required improvement score of Task Allocation is as follows:.

**FIGURE 5:**
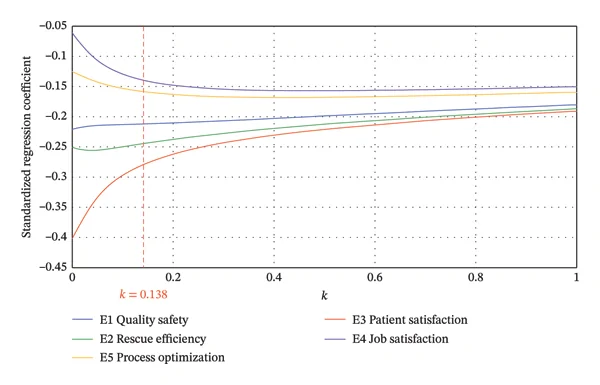
Ridge trace plot for the Task Allocation (O6) feedback equation. Note: The horizontal axis represents the ridge parameter *k*, and the vertical axis represents the standardized ridge regression coefficients. The curves show the trajectories of the standardized coefficients for Quality Safety, Rescue Efficiency, Patient Satisfaction, Job Satisfaction, and Process Optimization as *k* changes. The vertical dashed line indicates the selected ridge parameter, *k* = 0.138. When *k* approached 0.138, the signs, relative magnitudes, and trajectories of the coefficients began to stabilize. Therefore, 0.138 was selected as the ridge parameter to balance the reduction in multicollinearity against the introduction of estimation bias.

Task Allocation Required Improvement Score = 15.676 − 0.029 × Quality Safety − 0.014 × Job Satisfaction − 0.039 × Rescue Efficiency − 0.039 × Patient Satisfaction − 0.019 × Process Optimization.

From the above equation, it can be seen that, within the model, the required improvement score of Task Allocation is related to Quality Safety, Job Satisfaction, Rescue Efficiency, Patient Satisfaction, and Process Optimization.

#### 3.4.3. Model Testing

A preliminary internal evaluation was conducted to assess the structural interpretability and qualitative behavioral plausibility of the SD model. This study implemented model validation in two parts: the Structural Adaptability Test and Stability Test. The Structural Adaptability Test primarily focused on the scientificity and rationality of the model’s variables, causal relationships, and feedback structures. It included verifying the practical explanatory power of stocks, flows, and auxiliary variables in the nursing work performance system; confirming the consistency of the direction and logic of causal chains; ensuring the closure of key positive and negative feedback loops (in line with the dynamic laws of the system); and evaluating whether variable settings were concise and could cover the main influencing factors of the system. The Stability Test, through parameter sensitivity analysis and scenario simulation, assessed whether the model operated stably under different scenarios, and whether the output results were consistent with the expected behavior of the real system when disturbances to key parameters occurred.

In the formal validation phase, the causal loop diagram, stock‐and‐flow diagram, and simulation results of the model were provided to each expert, along with detailed explanations. The experts evaluated the model in terms of structural integrity, rationality of causal relationships, closure of feedback loops, rationality of boundary settings, and operational stability, in accordance with preset validation dimensions and criteria.

All four evaluators agreed that: (1) within the defined nursing unit boundary, the main variables were practically relevant, and the stocks, flows, and auxiliary variables were generally interpretable; (2) the directions of the main causal relationships and the closure of the feedback loops were broadly consistent with the nursing management mechanisms assumed in the model; (3) the system boundary and coverage of major factors were generally acceptable for the exploratory purpose of this study; and (4) under the parameter perturbation conditions presented to the experts, the directions of change in the main model outputs were considered qualitatively plausible and broadly consistent. None of the evaluators proposed revisions requiring changes to the fundamental model structure.

#### 3.4.4. Additional Verification Results

All equations passed symbolic dimensional consistency testing conducted independently of Vensim. Over the 12‐month simulation period, the reference run and all 32 implementation‐proportion scenarios remained within the assumed 0–100 range, with a maximum organizational‐factor stock value of 89.7. However, in the reference run extended to 120 months, the first exceedance of 100 by an organizational‐factor stock occurred at month 25.5, indicating that the original equations lacked an endogenous mechanism for limiting stock values.

At simulation Month 12, the central 95% scenario intervals for the composite nursing work performance index under nominal joint parameter perturbations of ±10% and ±20% were 54.0–63.3 and 48.7–67.3, respectively. No boundary violations occurred in the 5000 simulations within the ±10% perturbation range. Within the ±20% perturbation range, 660 of the 5000 simulations exceeded the assumed bounds, accounting for 13.2%. All simulations were retained, and boundary violations were flagged. The relative rankings of the five performance indices changed across the joint parameter simulations, indicating that comparisons among these indices were sensitive to the joint parameter assumptions.

#### 3.4.5. Model Simulation

This section simulates the change trajectories of nursing work performance over the following 12 months using the developed SD model. At the start of the simulation, the seven organizational‐factor stocks were initialized at 0, representing no accumulated organizational change relative to the simulation reference. The implementation fraction in each organizational‐factor accumulation‐rate equation was set to 0.5, and the simulation was run in Vensim PLE. The output is presented in Figure 6.

**FIGURE 6:**
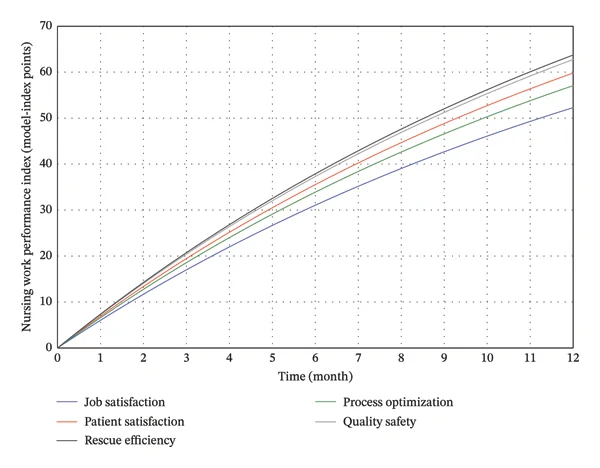
Twelve‐month simulation trajectories of the five types of nursing work performance under the reference run. Note: At the start of the simulation, the initial values of the seven organizational‐factor stocks were all set to 0, representing no accumulated organizational change relative to the simulation reference. The remaining parameters were set to their model‐estimated values, and no additional adjustments were applied to the organizational factors. The simulation period was 12 months, with a time step of 0.0625 months. The horizontal axis represents simulation time in months, and the vertical axis represents the dimensionless nursing work performance model index on the model’s native 0–100 scale. The five curves represent Quality Safety, Rescue Efficiency, Patient Satisfaction, Job Satisfaction, and Process Optimization. The simulation was conducted using Vensim PLE 10.3.1.

Figure 6 illustrates the changing trajectories of the five types of nursing work performance based on simulation analysis over a period of 1 year. The horizontal axis represents months, and the vertical axis represents the improvement level of nursing work performance. With regard to the overall trend, under the influence of the model’s reinforcing feedback, the aggregate curve exhibits an upward trend, suggesting that proactive adjustments and interventions at the organizational level influence individual factors, thereby effectively enhancing nursing work performance. With regard to performance‐type differences, a comparison of each performance type’s trajectory reveals distinct improvement levels.

The improvement in Rescue Efficiency was the greatest, reaching 63.7%. In the actual clinical environment, this means that when facing emergency rescue situations, nursing staff can respond more quickly and efficiently. The improvement in Quality Safety was 62.8%. This indicates that in actual nursing work, nursing staff pay more attention to operation norms and Quality Safety. Patient Satisfaction increased by 59.8%. This shows that there has been a significant improvement in the communication and interaction between nursing staff and patients. The improvement in Process Optimization was 57.1%. This means that the nursing workflow has been further optimized and improved. The improvement in Job Satisfaction was the smallest, at 52.3%. This reflects that, in the actual clinical environment, improving Job Satisfaction of nursing staff faces greater challenges. Job Satisfaction is not only determined by the work itself, but is also affected by various factors, such as work environment, interpersonal relationships, and career development. Therefore, medical institutions need to pay more attention to the Psychological State and career development needs of nursing staff and take effective measures to improve their Job Satisfaction.

## 4. Discussion

Nursing is the most prevalent occupation in medical institutions, and the nursing work performance not only relates to the efficiency of medical operations, but also closely affects patient safety. The nursing work performance is not only a focus of social attention, but also an academic hotspot. The nursing work performance has been actively discussed in previous studies. For instance, Quesada‐Puga et al. (2024) studied the impact of work environment and social support on nurses’ Job Satisfaction [36]. Adynski et al. studied the influence of organizational conditions, such as workload, support, material resources, and a healthy working environment on nursing work performance [12]. Prasetya et al. studied the impact of personal characteristics, such as gender, work experience, and nursing ability on the nursing work performance [11]. Khatatbeh et al. (2022) found that burnout level has an impact on work efficiency and service quality, and this can be alleviated by adjusting the workload at the organizational level [13]. Zumriyah et al. found that for every 1‐point increase in nursing work performance, Patient Satisfaction increases by an average of 0.8 points [37]. Li et al. studied the impact of nurses’ burnout level and nurse staffing on healthcare quality and Patient Satisfaction [14]. In summary, previous studies have explored the nursing work performance and its influencing factors from different perspectives based on questionnaire data, but they mainly focused on point‐to‐point discussions of a specific aspect, and there is less systematic exploration of the nursing work performance and its influencing factors. In response to the insufficient systematic exploration of the nursing work performance in previous studies, this study used the SD method to explore the mechanism of the impact between the nursing work performance and its influencing factors.

Improving nursing work performance requires a systems‐level perspective, as changes typically emerge from the synergistic action of multiple factors rather than from any single driver. First, this study addresses issues in prior research regarding the singular focus on nursing work performance types and the conflation and lack of systematization of influencing factors [38–40]. To overcome these issues, we conducted a systematic literature review, applying screening criteria to 2289 retrieved articles. Applying principles of unified term expression and clarifying meaning boundaries to these preliminary findings, we ultimately synthesized five types of nursing work performance, seven organizational factors, and three individual factors. This systematic categorization not only resolves prior inconsistencies in the expression of nursing work performance but also facilitates comparison across studies and a comprehensive understanding of nursing work performance and its determinants.

Second, clarifying how influencing factors affect nursing work performance is critical for healthcare institutions to develop more scientific and effective improvement strategies. However, previous research in this area has been mainly qualitative, failing to comprehensively incorporate all relevant factors [41], making it difficult to support effective managerial interventions. Therefore, using SD, we constructed causal loop and stock‐and‐flow diagrams encompassing organizational factors, individual factors, and nursing work performance, and quantitatively analyzed their dynamic interrelationships. Dynamic simulation provides a structured way to depict how these factors may interact and evolve over time, offering a systems‐level understanding of potential performance development and informing scenario‐based intervention discussions.

Below, we demonstrate how the SD model of nursing work performance and its influencing factors can inform priorities for future empirical testing for nursing departments. Specifically, the reference run used an implementation fraction of 0.5. In the alternative scenarios, it was set to 0.4 or 0.6, representing absolute changes of −0.1 or +0.1, and to 0.3 or 0.7, representing absolute changes of −0.2 or +0.2, while the remaining settings were held constant. Table 6 presents the simulated changes in nursing work performance levels.

**TABLE 6:** Twelve‐month simulation results for nursing work performance under organizational‐factor adjustment scenarios.

Simulation scheme	Implementation fraction	Month‐12 nursing work performance index (difference from the reference run, index points)				
		Quality Safety	Rescue Efficiency	Patient Satisfaction	Job Satisfaction	Process Optimization
Reference run	0.5	62.8 (−)	63.7 (−)	59.8 (−)	52.3 (−)	57.1 (−)

Cultural climate	0.4	62.5 (−0.3)	63.5 (−0.3)	59.5 (−0.3)	51.9 (−0.4)	56.7 (−0.3)
	0.3	62.2 (−0.6)	63.2 (−0.5)	59.2 (−0.6)	51.5 (−0.8)	56.4 (−0.7)
	0.6	63.0 (+0.3)	64.0 (+0.3)	60.1 (+0.3)	52.7 (+0.4)	57.4 (+0.3)
	0.7	63.3 (+0.6)	64.2 (+0.5)	60.4 (+0.6)	53.1 (+0.8)	57.7 (+0.7)

Leader attitude	0.4	62.3 (−0.5)	63.3 (−0.4)	59.3 (−0.5)	51.6 (−0.7)	56.5 (−0.5)
	0.3	61.9 (−0.9)	62.9 (−0.8)	58.8 (−1.0)	50.9 (−1.3)	56.0 (−1.1)
	0.6	63.2 (+0.4)	64.1 (+0.4)	60.3 (+0.5)	53.0 (+0.7)	57.6 (+0.5)
	0.7	63.7 (+0.9)	64.6 (+0.8)	60.8 (+1.0)	53.6 (+1.3)	58.2 (+1.1)

Supervisory governance	0.4	62.5 (−0.2)	63.5 (−0.2)	59.5 (−0.3)	51.9 (−0.4)	56.8 (−0.3)
	0.3	62.3 (−0.5)	63.3 (−0.5)	59.3 (−0.5)	51.6 (−0.7)	56.5 (−0.6)
	0.6	63.0 (+0.2)	64.0 (+0.2)	60.1 (+0.3)	52.7 (+0.4)	57.4 (+0.3)
	0.7	63.3 (+0.5)	64.2 (+0.5)	60.4 (+0.5)	53.0 (+0.7)	57.7 (+0.6)

Incentive support	0.4	61.5 (−1.3)	62.6 (−1.1)	58.4 (−1.4)	51.2 (−1.1)	55.9 (−1.2)
	0.3	60.2 (−2.6)	61.5 (−2.2)	56.9 (−2.9)	50.1 (−2.2)	54.7 (−2.4)
	0.6	64.0 (+1.3)	64.8 (+1.1)	61.2 (+1.4)	53.3 (+1.0)	58.2 (+1.2)
	0.7	65.3 (+2.5)	65.8 (+2.1)	62.6 (+2.7)	54.4 (+2.1)	59.4 (+2.3)

Resource allocation	0.4	61.9 (−0.9)	62.6 (−1.1)	59.2 (−0.6)	51.5 (−0.8)	56.3 (−0.8)
	0.3	61.0 (−1.7)	61.5 (−2.2)	58.5 (−1.3)	50.7 (−1.6)	55.5 (−1.6)
	0.6	63.6 (+0.8)	64.8 (+1.1)	60.4 (+0.6)	53.1 (+0.8)	57.9 (+0.8)
	0.7	64.4 (+1.7)	65.9 (+2.1)	61.1 (+1.2)	53.8 (+1.5)	58.6 (+1.6)

Task allocation	0.4	60.2 (−2.5)	61.0 (−2.7)	57.6 (−2.2)	50.4 (−1.9)	54.9 (−2.2)
	0.3	57.6 (−5.2)	58.1 (−5.6)	55.3 (−4.6)	48.5 (−3.8)	52.6 (−4.5)
	0.6	65.2 (+2.4)	66.4 (+2.6)	61.9 (+2.1)	54.1 (+1.8)	59.1 (+2.1)
	0.7	67.5 (+4.7)	68.9 (+5.1)	64.0 (+4.2)	55.8 (+3.5)	61.1 (+4.1)

Education training	0.4	59.9 (−2.9)	60.9 (−2.8)	57.0 (−2.8)	50.3 (−2.0)	54.7 (−2.4)
	0.3	56.9 (−5.9)	57.9 (−5.8)	54.1 (−5.7)	48.3 (−4.0)	52.1 (−5.0)
	0.6	65.5 (+2.7)	66.4 (+2.7)	62.5 (+2.6)	54.1 (+1.9)	59.4 (+2.3)
	0.7	68.1 (+5.3)	69.0 (+5.2)	65.0 (+5.1)	55.9 (+3.6)	61.5 (+4.5)

Overall change	0.4	53.7 (−9.1)	54.5 (−9.2)	51.2 (−8.6)	44.7 (−7.6)	48.8 (−8.2)
	0.3	43.2 (−19.6)	43.8 (−19.9)	41.1 (−18.7)	36.0 (−16.3)	39.2 (−17.8)
	0.6	70.5 (+7.8)	71.6 (+7.9)	67.2 (+7.4)	58.8 (+6.5)	64.1 (+7.1)
	0.7	77.2 (+14.4)	78.4 (+14.7)	73.6 (+13.8)	64.4 (+12.1)	70.2 (+13.1)

*Note:* The reference run used an implementation fraction of 0.5 in all seven organizational‐factor accumulation‐rate equations. In the alternative scenarios, the implementation fraction was set to 0.3, 0.4, 0.6, or 0.7 for one organizational factor at a time or for all seven factors simultaneously, while the other model settings were held constant. Values outside parentheses are the Month‐12 model‐index values, whereas values in parentheses are index‐point differences from the reference run. All simulated values are dimensionless, assumption‐dependent model outputs generated under the specified model structure and parameter settings. They have not been externally validated using real‐world longitudinal data and should not be interpreted as observed clinical improvements, causal effect estimates, or precise predictions.

From Table 6, the following observations can be drawn:

1. When all seven organizational factors are adjusted positively together, the magnitude of change across the five types of nursing work performance exceeds that achieved by adjusting any single organizational factor. This finding is consistent with previous research emphasizing that nursing work performance is jointly influenced by multiple organizational factors. For example, based on nurse survey data from 665 healthcare institutions in the United States, Aiken et al. reported that coordinated improvements in staffing, Education and Training, and a supportive work environment produced greater benefits for patient safety and quality of care [42]. Through a systematic review, Paguio et al. proposed that the integrated implementation of multiple strategies, including leadership engagement, nurse empowerment, communication and collaboration, and educational support, could more effectively improve Job Satisfaction, quality of care, and organizational effectiveness [43].
 Extending previous research that primarily emphasized the effectiveness of implementing multiple organizational measures in combination, the present study incorporated seven organizational factors into a unified SD model. By comparing the simulation results of single‐factor adjustments with those of the simultaneous adjustment of multiple factors, this study demonstrated the potential cumulative and synergistic effects among organizational factors. This extension not only suggests that improvements in nursing work performance should not rely on a single management measure but also provides an analytical framework for comparing the potential effects of integrated and individual interventions.
2. Even when adjusting organizational factors as a whole, the magnitudes of change differ by performance type. For example, increasing the implementation fractions for all seven organizational factors from 0.5 to 0.7 yields the greatest gains in Rescue Efficiency (14.7 index points) and Quality Safety (14.4 index points), followed by Patient Satisfaction (13.8 index points) and Process Optimization (13.1 index points), with Job Satisfaction improving the least (12.1 index points). This suggests that Rescue Efficiency and Quality Safety are relatively more responsive, whereas Job Satisfaction requires longer term accumulation to change. This finding is broadly consistent with previous evidence that different nursing outcomes respond differently to changes in organizational factors. For example, after systematically reviewing longitudinal studies, Dall’Ora et al. found relatively consistent evidence that higher registered nurse staffing levels reduced the risk of patient mortality, whereas the magnitude and consistency of evidence for other outcomes, including falls, infections, pressure injuries, and readmissions, were comparatively weaker [44]. This finding indicates that different performance indicators may respond to the same organizational factor to different degrees. By comparing outcomes from 27 intervention hospitals and 28 control hospitals before and after the implementation of minimum nurse staffing standards in Queensland, Australia, McHugh et al. found that improvements in nurse staffing were associated with significant reductions in patient mortality, readmission rates, and length of stay [45]. These findings suggest that staffing adjustments may be reflected relatively quickly in direct clinical outcomes, such as Rescue Efficiency and Patient Safety. After systematically reviewing 28 studies conducted in long‐term care facilities, Aloisio et al. reported that nurses’ Job Satisfaction was jointly influenced by multilevel factors, including autonomy, psychological empowerment, work stress, burnout, management support, staffing, and interpersonal relationships [46]. Job Satisfaction is therefore a subjective occupational outcome shaped cumulatively by multiple factors, and its improvement may depend on the sustained effects of several organizational conditions. Consequently, its short‐term magnitude of change may be smaller than that of more direct performance outcomes, such as Rescue Efficiency and Quality Safety.
 Conversely, when the implementation fractions for all seven organizational factors were reduced from 0.5 to 0.3, Job Satisfaction showed the smallest modeled decrease, whereas Rescue Efficiency and Quality Safety showed the largest decreases. Rescue Efficiency increased by 14.7 index points under the 0.7 implementation‐fraction scenario but decreased by 19.9 index points under the 0.3 scenario. The above results demonstrate that even within the category of work performance, their change mechanisms differ. Although previous studies have rarely examined why negative changes may be greater than positive changes of the same magnitude, this simulation result can be partially explained by research on occupational burnout. A systematic review and meta‐analysis by Li et al. showed that Quality Safety, patient experience, and other nursing outcomes may differ in their sensitivity to the same organizational pressures or staffing conditions [14]. Therefore, when workload increases, staff support decreases, or the work environment deteriorates, occupational burnout may act as an intermediate mechanism that amplifies the adverse effects of deteriorating organizational conditions on nursing work performance. Nevertheless, the asymmetric changes identified in this study—that is, the differences between increases and decreases in organizational factors—were derived from model‐based scenario simulations. They should not be interpreted as causal effects validated using real‐world longitudinal data but rather as dynamic hypotheses requiring further empirical examination.
 Previous studies have generally examined individual outcomes, such as mortality, readmission, Job Satisfaction, or patient experience, separately. In contrast, the present study compared the magnitude of change across five types of nursing work performance under the same organizational‐factor adjustment scenarios, thereby further revealing differences in their sensitivity and potential speed of response. The present study also compared the simulation results of equivalent increases and decreases in organizational factors and proposed that nursing work performance may respond asymmetrically to improvements and deterioration in organizational conditions. This finding extends the existing literature from determining whether organizational factors affect performance to examining how different types of performance respond and whether their responses to positive and negative changes are symmetrical.
3. Each organizational factor impacts work performance types differently. When the implementation fraction for each organizational factor was individually increased from 0.5 to 0.7, Education and Training and Task Allocation yield improvements in the range of 3.5–5.3 index points across the five performance types, whereas Cultural Climate and Supervisory Governance produce more modest gains of 0.5–0.8 index points. This finding is broadly consistent with previous evidence that different organizational factors have distinct pathways and magnitudes of influence. For example, through a systematic review and meta‐analysis of educational interventions for evidence‐based practice among clinical nurses, Sapri et al. found that Education and Training significantly improved nurses’ evidence‐based knowledge, skills, attitudes, and practice behaviors [47]. This evidence suggests that training can directly strengthen the professional competencies required to perform nursing tasks and may therefore have relatively rapid effects on Quality Safety, process efficiency, and patient outcomes. Twigg et al. systematically reviewed the effects of different nurse staffing methods on nurse and patient outcomes and proposed that appropriate staffing and task assignment could directly alter the time and resources available to nurses, thereby exerting broad effects on multiple performance outcomes [48]. Using structural equation modeling to analyze nurse survey data, Tate et al. found that authentic leadership and a developmental organizational culture primarily improved nursing quality and Patient Satisfaction indirectly by promoting hospital quality management practices [49]. This finding indicates that Cultural Climate and governance‐related factors may affect performance through longer pathways and may therefore have relatively limited direct effects in the short term.
 Moreover, when the Cultural Climate implementation fraction was increased from 0.5 to 0.7, Job Satisfaction shows the largest growth among the five types of nursing work performance; when the Incentives and Support implementation fraction was increased from 0.5 to 0.7, Patient Satisfaction exhibits the highest growth; and when the Task Allocation implementation fraction was increased from 0.5 to 0.7, Rescue Efficiency achieves the largest growth. This indicates that there are differences in the effectiveness of different organizational factors in improving nursing work performance. They are also consistent with previous evidence that organizational factors may have differential strengths in influencing specific nursing outcomes. For example, a literature review by Lu et al. found that nurses’ Job Satisfaction was closely associated with organizational culture, the work environment, organizational commitment, structural empowerment, and team social capital [50]. This finding indicates that a supportive, collaborative, and equitable organizational climate primarily influences nurses’ subjective evaluations of their work environment and professional value. The systematic review by Jun et al. similarly reported that lower nurse burnout and more positive occupational states were associated with better patient experiences [15]. It can therefore be inferred that Incentives and Support may exert a particularly strong influence on Patient Satisfaction by increasing nurses’ work engagement, reducing emotional exhaustion, and improving nurse–patient interactions. After systematically reviewing longitudinal studies, Dall’Ora et al. also found that higher registered nurse staffing levels were generally associated with a lower risk of patient mortality [44].
 Building on previous studies that separately examined Education and Training, staffing, organizational culture, or leadership support, the present study compared the relative effects of seven organizational factors within a unified model and under the same adjustment magnitude. The findings identified factors with broader and stronger short‐term effects, such as Education and Training and Task Allocation, and further indicated that Cultural Climate, Incentives and Support, and Task Allocation may exert relatively stronger effects on Job Satisfaction, Patient Satisfaction, and Rescue Efficiency, respectively. Thus, this study extends the fragmented evidence on individual organizational factors into a systematic comparison across organizational factors and performance types and further distinguishes differences in both the scope and magnitude of their effects.

### 4.1. Implications for Nursing Management

SD models are primarily used to reveal feedback relationships, time delays, and potential policy consequences; they cannot independently demonstrate that a specific management measure will necessarily produce the effects shown by the model [51, 52]. Because the present model has not been externally calibrated or validated using real‐world longitudinal data, its implications for nursing management should be interpreted as priorities for empirical testing. Table 6 shows that, under the current model structure and parameter settings, coordinated improvements in organizational factors may produce a greater overall effect than adjustments to a single factor. It also shows that different types of nursing work performance vary in their sensitivity to changes in organizational factors and that different organizational factors may have differential effects on specific performance types. Based on these findings, this study proposes the following four priorities for empirical testing:

1. Examine differences between coordinated changes in multiple factors and changes in a single factor
 The model results show that when all seven organizational factors were improved simultaneously, the magnitude of change in all five types of nursing work performance was greater than that produced by adjusting a single factor. This suggests that nursing work performance may be jointly influenced by Education and Training, Task Allocation, Resource Allocation, Incentives and Support, Leader Attitude, Supervisory Governance, and Cultural Climate. Future longitudinal studies, quasi‐experimental studies, or controlled pilot studies could compare the actual effects of individual organizational measures with those of combinations of multiple organizational measures. These studies should measure both actual changes in organizational conditions and corresponding changes in the five categories of nursing work performance to determine whether the cumulative effects indicated by the model can be replicated in real‐world nursing settings.
2. Examine asymmetric responses to improvements and deterioration in organizational conditions
 The model results show that the performance losses caused by an overall deterioration in organizational factors were greater than the gains produced by improvements of the same magnitude, with Rescue Efficiency and Quality Safety being particularly sensitive to negative changes. Future research could use longitudinal studies, natural experiments, or interrupted time‐series designs to examine nursing work performance before and after changes in staffing, workload, Education and Training, or resource conditions. Until this relationship has been empirically validated, simulation results should not be used to establish universal early‐warning thresholds or assess actual management risk levels.
3. Validate organizational factors associated with different performance outcomes
 Sensitivity analysis identified Education and Training and Task Allocation as candidate factors warranting priority testing across multiple performance outcomes. It also generated more specific hypotheses regarding potential relationships between Cultural Climate and Job Satisfaction, Incentives and Support and Patient Satisfaction, and Task Allocation and Rescue Efficiency. Future studies should prespecify these hypotheses, use validated measurement instruments, and examine whether the direction and relative strength of these relationships remain consistent across hospitals, nursing units, and clinical settings. Until empirical validation has been completed, interventions should not be selected, nor Resource Allocation decisions made, solely on the basis of the rankings from the current sensitivity analysis.
4. Collect multicenter longitudinal data for model calibration and external validation
 Future studies should repeatedly measure organizational factors, nurses’ Physiological State, Psychological State, and Professional Competence, and the five categories of nursing work performance across different hospitals and nursing units. These real‐world data should be used to estimate and recalibrate model parameters, test the time delays and feedback relationships specified in the model, and compare simulated trends with observed outcomes. Only after the model demonstrates acceptable calibration and external validity should its potential use as a management decision‐support tool be further evaluated.

## 5. Conclusion

Through a systematic literature review, this study synthesized and defined five types of nursing work performance, seven organizational factors, and three individual factors from 47 included studies and developed a SD model describing the relationships among organizational factors, individual factors, and nursing work performance. The results indicate that organizational factors are associated with nurses’ Physiological State, Psychological State, and Professional Competence and influence nursing work performance through these individual factors. Changes in nursing work performance also exert feedback effects on organizational factors. Under the specified implementation‐fraction scenarios, simultaneously setting the implementation fractions of all seven organizational factors to 0.7 produced larger model‐index changes than changing the fraction for any single factor to 0.7. Relative to the reference run at 0.5, the corresponding Month‐12 differences were 14.7 index points for Rescue Efficiency, 14.4 for Quality Safety, 13.8 for Patient Satisfaction, 13.1 for Process Optimization, and 12.1 for Job Satisfaction, respectively. Rescue Efficiency and Quality Safety were relatively sensitive to organizational adjustments, whereas the improvement in Job Satisfaction was comparatively modest, suggesting that it may require more sustained organizational support and longer term psychological accumulation. The effects also varied across organizational factors. Education and Training and Task Allocation generally exerted stronger positive effects across the performance types; Cultural Climate had a particularly strong influence on Job Satisfaction; Incentives and Support produced a comparatively larger improvement in Patient Satisfaction; and Task Allocation had a more pronounced effect on Rescue Efficiency.

The main contribution of this study is the integration of previously fragmented types of nursing work performance and their organizational‐ and individual‐level determinants into a unified analytical framework. The SD approach further revealed the feedback, cumulative, and temporal mechanisms operating among these multilevel factors. Compared with studies that examine static relationships between a single factor and a single performance outcome, the present study provides a dynamic systems perspective for comparing the potential changes in nursing work performance under different combinations of organizational adjustments. Its primary value is therefore to generate empirically testable hypotheses and identify priorities for future nursing management research, rather than to prescribe specific management interventions. All simulated values are dimensionless, assumption‐dependent model outputs generated under the specified model structure and parameter settings. They have not been externally validated using real‐world longitudinal data and should not be interpreted as observed clinical improvements, causal effect estimates, or precise predictions.

This study has several limitations. First, the model focuses on the nursing work subsystem at the level of a single nursing department and does not fully incorporate cross‐departmental factors involving physicians, other healthcare professionals, or the broader institutional environment of the hospital. Second, model development and parameterization were primarily based on evidence from the literature and expert judgments. The sample size and sources of expert input may limit the applicability of the model across different regions, hospital types, and nursing settings. Third, the model has not yet been externally calibrated or validated using real‐world nursing work performance data. Future studies should therefore apply the model in actual nursing settings and collect longitudinal nursing work performance data to calibrate, externally validate, and further optimize the model. In other words, comprehensive and rigorous validation and refinement using real‐world data will be required before the model can be applied in practice. In addition, although this study simulated multiple adjustment scenarios, specific implementation procedures—such as training program design and organizational restructuring—and cost‐effectiveness analyses require further investigation.

## Funding

This work was supported by the National Natural Science Foundation of China (Grant Nos. 72171042 and 72671061) and the Fundamental Research Funds for the Central Universities (Grant No. N2406006).

## Disclosure

The funding agency had no role in the study design, data collection, data analysis, manuscript preparation, or decision to publish.

## Conflicts of Interest

The authors declare no conflicts of interest.

## Supporting Information

Additional supporting information can be found online in the Supporting Information section.

## Supporting information


**Supporting Information 1** Completed PRISMA 2020 Checklist. This file provides the completed PRISMA 2020 checklist and identifies the manuscript page corresponding to each applicable reporting item.

**Supporting Information 2** Study‐Level JBI Critical Appraisal. This Excel file provides the complete item‐level JBI critical appraisal results for all 47 included studies.

**Supporting Information 3** Evidence‐to‐Pathway Mapping. This Excel file maps all 47 included studies to the three model pathways: R1, organizational factors to individual factors; R2, individual factors to nursing work performance; and R3, nursing work performance to organizational factors.

**Supporting Information 4** Reproducible Vensim Model. This unit‐annotated Vensim model file contains the executable model equations, initial values, parameter settings, and simulation controls used to reproduce the model structure and reference‐run simulations.

**Supporting Information 5** System Dynamics Model Equations and Simulation Parameters. This file contains the variables, equations, initial conditions, and simulation control parameters for the system dynamics model implemented in Vensim PLE 10.3.1.

## Data Availability

The data that support the findings of this study are available upon request from the corresponding author. The data are not publicly available due to privacy or ethical restrictions.
